# Danggui Buxue Decoction Sensitizes the Response of Non-Small-Cell Lung Cancer to Gemcitabine via Regulating Deoxycytidine Kinase and P-glycoprotein

**DOI:** 10.3390/molecules24102011

**Published:** 2019-05-25

**Authors:** Xiyang Sun, Xin Xu, Yanfei Chen, Rong Guan, Tingting Cheng, Ye Wang, Rui Jin, Min Song, Taijun Hang

**Affiliations:** 1 Key Laboratory of Drug Quality Control and Pharmacovigilance of the Ministry of Education, China Pharmaceutical University, Nanjing 210009, China; donkey_ever@126.com (X.S.); xuxin_06_04@163.com (X.X.); cyf19920915@163.com (Y.C.); cpuguanrong@126.com (R.G.); ttcheng13@163.com (T.C.); 1721010159@stu.cpu.edu.cn (Y.W.); 1721010176@stu.cpu.edu.cn (R.J.); 2 Department of Pharmaceutical Analysis, China Pharmaceutical University, Nanjing 210009, China

**Keywords:** Danggui Buxue decoction, TCM formula, gemcitabine, pharmacokinetics, herb–drug interaction, deoxycytidine kinase, P-glycoprotein

## Abstract

This study aimed to investigate whether the anti-tumor effect of gemcitabine (GEM) in non-small-cell lung cancer (NSCLC) treatment was affected by Danggui Buxue decoction (DBD), and explore the potential mechanisms. The combined use of GEM and DBD showed an enhanced tumor growth inhibition effect in a murine Lewis lung carcinoma (LLC) model. LC-MS/MS results showed that the pharmacokinetic behaviors of a GEM active metabolite, gemcitabine triphosphate (dFdCTP), were found to be altered remarkably in the peripheral blood mononuclear cells (PBMC) of DBD co-administration rats. In addition, after co-administration of DBD with GEM, Western Blot and qPCR results confirmed that the expression of deoxycytidine kinase (dCK) in tumor tissues of LLC-bearing mice were markedly increased. DBD co-administration also reversed the upregulation of P-glycoprotein (P-gp) in tumor tissues induced by GEM. Moreover, DBD could notably up-regulate the IL-12p70 and GM-CSF expression in mice serum, suggesting potential immunomodulatory activities in tumor-bearing mice. Meanwhile, DBD inhibited the P-gp efflux activity in A549 cells. Therefore, the regulation of dCK and P-gp played important roles in the alternation of GEM pharmacokinetics and the enhancement of the anti-tumor effect of GEM. DBD being a potential dCK promoter could work as an adjuvant agent to boost the anticancer effect of GEM.

## 1. Introduction

Lung cancer is the leading cause of cancer death all over the world. There were an estimated 2,093,876 new cases and 1,761,007 deaths all over the world in 2018 [1]. Non-small-cell lung cancer (NSCLC), which has a more variable behavior, accounts for about 85% of lung cancers. Only 18% of the patients suffering from lung cancer are expected to live five years after diagnosis [2].

Chemotherapy is currently the most effective remedy for NSCLC. However, despite the improved outcomes for NSCLC patients by various chemotherapeutic drugs, the growing drug resistance and severe side effects have been a major reason for the failure of chemotherapy [3].

Gemcitabine (GEM), a nucleoside analog also known as 2′,2′-Difluorodeoxycytidine (dFdC), is one of the most commonly used chemotherapeutic drugs in the treatment of NSCLC [4]. As a pro-drug, GEM has to enter the tumor cell to become active, and a major part of GEM is converted into the inactive metabolite 2′,2′-difluorodeoxyuridine (dFdU) by the deamination effect of cytidine deaminase (CDA). The cellular uptake of GEM is largely mediated by human equilibrative nucleoside transporter (hENT) and human concentrative nucleoside transporters (hCNT) [5,6]. Two active metabolites of GEM, gemcitabine diphosphate (dFdCDP) and gemcitabine triphosphate (dFdCTP), play an essential role against cancer [7]. dFdCDP blocks the nucleoside reductase, and therefore reduces the level of deoxynucleotide in cancer cells. During DNA replication, dFdCTP replaces one of the nucleosides to arrest the cell proliferation, resulting in apoptosis [8]. However, the growing chemo-resistance and side effects have restricted the clinical application of GEM [9]. Although several resistance mechanisms are involved in GEM metabolism, deoxycytidine kinase (dCK) is of particular interest because it is the rate-limiting enzyme in the phosphorylation process from dFdC to dFdCDP and dFdCTP [8].

Based on these issues caused by chemotherapies, some studies tried to apply traditional herbal medicines to the treatment of cancers [10,11,12]. According to these studies, some traditional herbal medicines were found to have the ability to increase the efficacy and reduce the side effects of chemotherapeutic drugs. Herbal remedies such as garlic, green tea, ginger, or noni juice were often reported in herb–drug combination therapy with pyrimidine analogues such as fluorouracil and gemcitabine, and platinum compounds such as carboplatin and oxaliplatin [13]. According to the literature, the pharmacokinetic interactions between traditional herb medicine and drugs are mainly caused by regulation of metabolic enzymes and drug transporters [14], especially for the efflux transporter P-gp. P-gp is a well-known obstacle of effective chemotherapy, and classic multidrug resistance is associated with the overexpression of P-gp, resulting in an increased efflux of chemotherapy drugs [15], and recent research has indicated that down-regulation of P-gp expression could increase gemcitabine sensitivity [16,17,18].

Danggui buxue decoction (DBD), a traditional Chinese medicinal decoction which consists of Radix *Astragalus membranaceus var. mongholicus (Bunge) P.K.Hsiao* (RA) and Radix *Angelica sinensis* (Oliv.) Diels (RAS) at a ratio of 5:1, which was first described in Neiwaishang Bianhuo Lun by Li Dongyuan in China, has been widely used in traditional Chinese medicine due to its extraordinary immune regulation and hematopoietic effect [19]. Clinical studies validated that DBD could elevate the immune function of an organism and improve the quality of life in NSCLC patients [20]. According to literature, an astragalus-based herbal formula showed potential to increase the effectiveness of platinum-based chemotherapy when combined with chemotherapy in NSCLC treatment [21]. Astragaloside IV, an active compound in astragalus, was found to be able to enhance cisplatin chemo-sensitivity in NSCLC cells through inhibition of CD276 [22]. However, little information is available about the combination effect of DBD and GEM. Meanwhile, the underlying mechanisms of the immunoregulation effects of DBD remain largely unknown.

In the present study, we compared the pharmacokinetic profiles of intravenous administrated GEM with or without oral co-administration of DBD in rat plasma and peripheral blood mononuclear cells (PBMC). In addition, by utilizing a Lewis lung carcinoma (LLC) murine model, we evaluated the anti-tumor effect of GEM altered by a combination administration of DBD and determined the mRNA and protein expression level of dCK and P-gp in tumor tissue of LLC model mice. The findings obtained from these results are expected to provide scientific basis for clarifying the mechanisms of action and combination of DBD in the treatment of NSCLC by GEM.

## 2. Results

### 2.1. Determination of the Combination Effect of DBD and GEM

**Figure 1:**
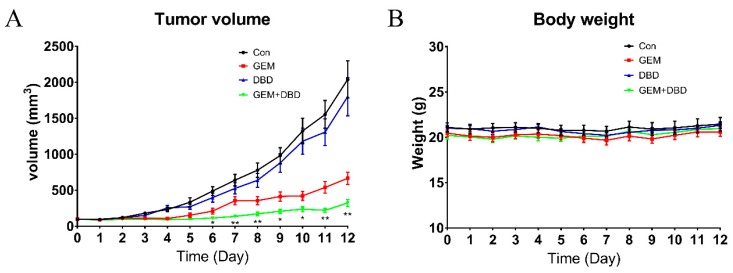
Tumor volume (**A**) and body weight (**B**) of Lewis lung carcinoma (LLC) tumor-bearing mice during 12 days of treatment. Data were expressed as mean ± SEM (*n* = 10). * *p* < 0.05, Gemcitabine (GEM) + Danggui Buxue decoction (DBD) versus GEM. ** *p* < 0.01, GEM + DBD versus GEM. Mice were administrated vehicle alone (saline), GEM alone (100 mg/kg i.p., once a week), DBD alone (1.8 g/kg i.g., each day), or a combination of GEM and DBD (same as the relevant single drug group).

To evaluate whether the tumor growth inhibition effects of GEM were enhanced by co-administration of DBD, an LLC mouse model was established by subcutaneous injection of LLC cells into the right flank of each C57BL/6 mouse. After co-administration of DBD and GEM, the weights (Figure 1B), behaviors, diets, and mental states of the mice showed no obvious abnormalities. The tumor volumes in the GEM group and GEM + DBD group were significantly decreased, compared with the control group (Figure 1A). Moreover, DBD combined with GEM treatment significantly reduced tumor volume compared with the GEM group, from day 6 to day 12. Meanwhile, DBD treatment exhibited no significant effect on tumor volume compared with the control group, and the body weights of all four groups showed no significant change.

### 2.2. In Vivo Plasma Comparative Pharmacokinetics

**Figure 2:**
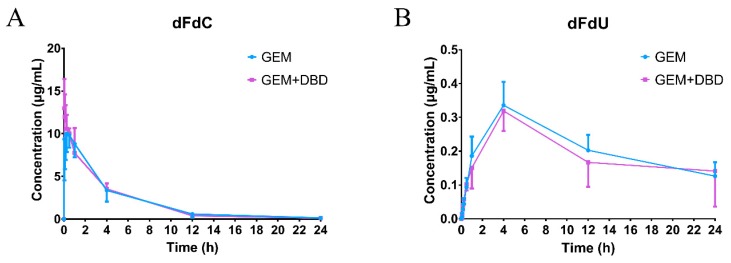
Concentration-time profiles of dFdC (**A**) and dFdU (**B**) in rat plasma after administration of GEM and DBD. Data were expressed as mean ± SD (*n* = 10). Rats in the GEM group received a tail vein bolus intravenous administration of 50 mg/kg gemcitabine. In the GEM + DBD group, DBD was co-administered at the dose of 3.6 g/kg by intra gavage administration, whereas the GEM group was treated with saline.

The validated LC-MS/MS method was successfully applied to the comparative pharmacokinetics study of dFdC and dFdU in rat plasma. The mean plasma concentration-time curves are shown in Figure 2. The major pharmacokinetic parameters are presented in Table 1. The pharmacokinetic parameters of dFdC and dFdU showed no significant difference between the GEM and GEM + DBD group.

### 2.3. In Vivo Peripheral Blood Mononuclear Cells (PBMC) Comparative Pharmacokinetics

**Figure 3:**
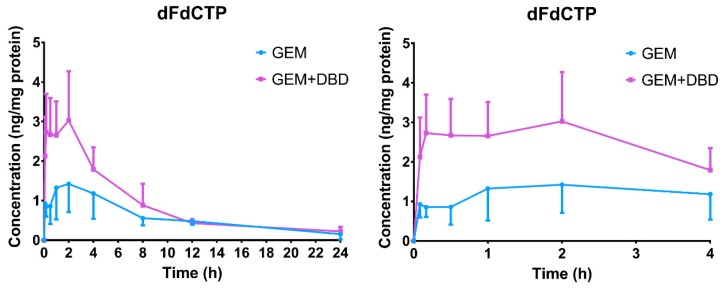
Concentration-time profiles of dFdCTP in rats peripheral blood mononuclear cells (PBMC) after administration of GEM and DBD (with partially enlarged view on the right). Data were expressed as mean ± SD (*n* = 10). Rats in the GEM group received a tail vein bolus intravenous administration of 50 mg/kg gemcitabine. In the GEM + DBD group, DBD was co-administered at the dose of 3.6 g/kg by intra gavage administration, whereas the GEM group was treated with saline.

The previously mentioned LC-MS/MS method [23] was successfully applied to the comparative pharmacokinetics study of dFdCMP, dFdCDP, and dFdCTP in rat PBMC. However, only the dFdCTP level was higher than the detection limit, while dFdCMP and dFdCDP levels were lower than the lower limit of quantification (LLOQ) of 0.21 ng/mL and 0.32 ng/mL, respectively. The mean plasma concentration-time curves are shown in Figure 3. The major pharmacokinetic parameters are presented in Table 2. The significant difference of T_max_ (*p* < 0.001) may be attributed to the metabolism acceleration of dFdCTP from dFdC. No significant difference of t_1/2_ was found between the GEM and the GEM + DBD groups, which gave us a clue that DBD may not affect the elimination of dFdCTP. The maximum dFdCTP concentration (C_max_) in the GEM + DBD group was found to be 3.80 ± 0.79 ng/mg protein, which was significantly higher (about 1.85-fold) than that of the GEM group (*p* < 0.001). The AUC_0–t_ of dFdCTP was found to be 1266 ± 145 ng·min/mg, which was increased by 1.59-fold as compared to the GEM group (*p* < 0.001).

### 2.4. Protein Expression of dCK and P-gp in Lewis Lung Carcinoma (LLC)-bearing Mice Tumor Tissue

**Figure 4:**
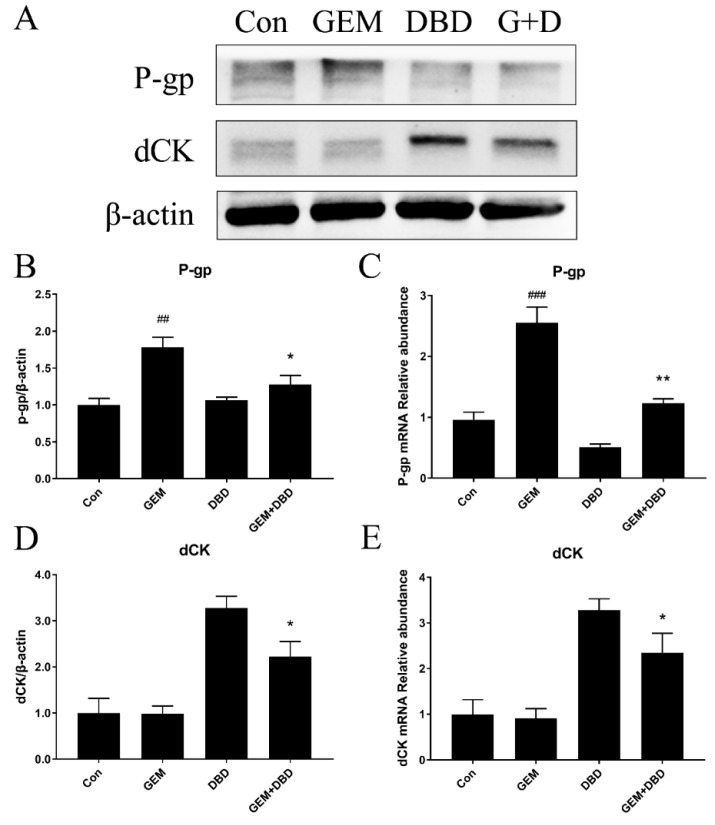
Expression of P-gp and dCK protein (**A**,**B**,**D**), and mRNA (**C**,**E**) level in tumor tissue of LLC tumor-bearing mice. Data were expressed as mean ± SEM (*n* = 10) * *p* < 0.05, GEM + DBD versus GEM. ** *p* < 0.01, GEM + DBD versus GEM. ^##^ *p* < 0.01, GEM versus control (Con). ^###^ *p* < 0.001, GEM versus Con.

Western blot analysis was performed and the relative expressions of P-gp and dCK in mice tumor tissue are shown in Figure 4A. It was found that after 12 days of DBD plus GEM treatment, the dCK level was significantly increased as compared with that of GEM group (Figure 4B). The P-gp expression level was obviously higher in the GEM group than in the control (Con) group, and the combination administration of DBD canceled the up-regulation effect (Figure 4C).

### 2.5. mRNA Expression of dCK and P-gp in Lewis Lung Carcinoma (LLC)-bearing Mice Tumor Tissue

P-gp and dCK mRNA expression in tumors was evaluated by real-time quantitative polymerase chain reaction (qPCR). As seen in Figure 4D,E, the level of dCK in the GEM + DBD group was significantly higher than that in the GEM group. While the level of P-gp in the GEM group was found to be up-regulated compared with that in the control group, which could be reversed by the combination administration of DBD (Figure 4B,C).

### 2.6. Immune Regulatory Effect of DBD on LLC-Bearing Mice

**Figure 5:**
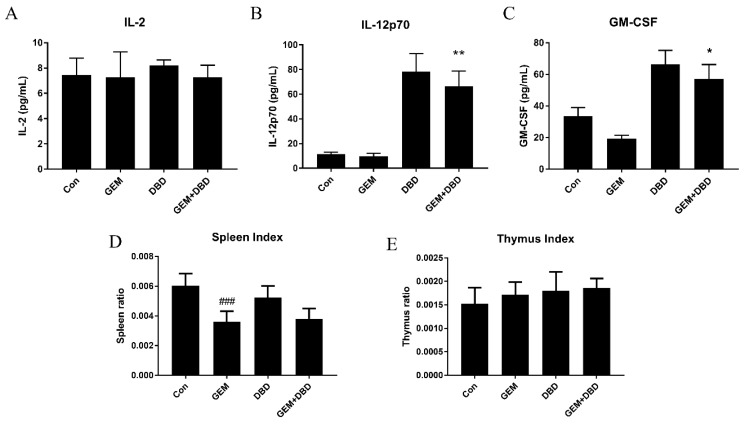
Immune regulatory effect of DBD on LLC-bearing mice. ELISA results showing IL-2 (**A**), IL-12 (**B**) and GM-CSF (**C**) expression in LLC tumor-bearing mice serum. (**D**) Spleen indexes and (**E**) Thymus indexes of tumor-bearing mice. Data were expressed as mean ± SEM (*n* = 4 for A, B, and C. *n* = 10 for D and E). * *p* < 0.05, GEM + DBD versus GEM. ** *p* < 0.01, GEM + DBD versus GEM. ^###^ *p* < 0.001, GEM versus Con.

The thymus and spleen indices can reflect the immune function and indicate the immune regulatory effect of DBD. As shown in Figure 5D,E, the spleen index of the GEM group mice decreased significantly when compared with the control group, and there was no significant difference between GEM and GEM + DBD mice in spleen and thymus indexes. To investigate cytokine level in LLC-bearing mice plasma, protein expression levels of IL-2, IL-12p70, and granulocyte-macrophage colony-stimulating factor (GM-CSF) were assessed by ELISA. IL-2 level between the GEM + DBD group and the GEM group were of no statistical significance (Figure 5A), while IL-12p70 and GM-CSF levels were significantly higher in the combination group (Figure 5B,C).

### 2.7. P-gp Efflux Activity Measurement by Rh 123 Accumulation Assay

**Figure 6:**
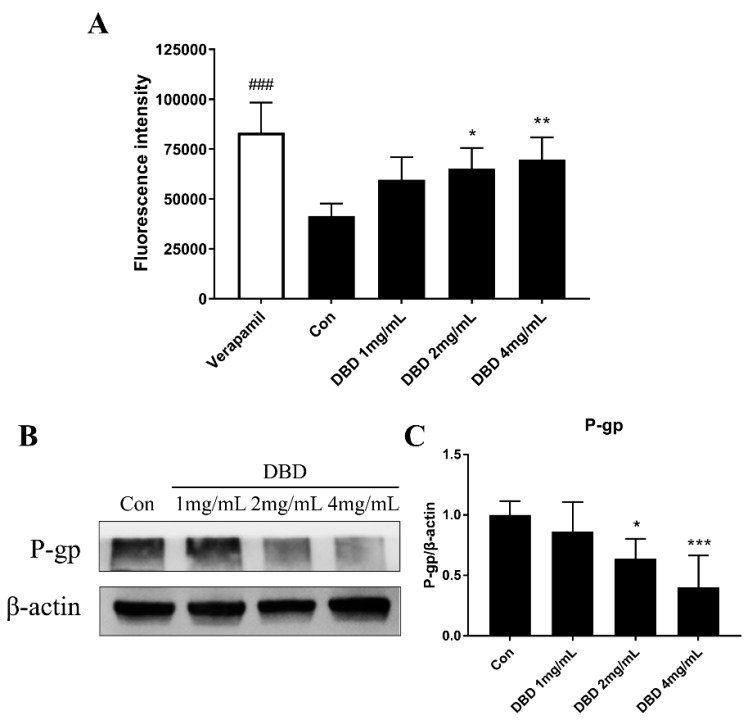
Effect of DBD on P-gp efflux activity and protein expression in A549 Cells. (**A**) The intracellular mean fluorescence intensity associated with Rh 123 was determined by flow cytometry. Verapamil (50 μM) was used as a positive control. (**B** and **C**) The protein expression of P-gp in A549 cells. Data were expressed as mean ± SD of five independent experiments. * *p* < 0.05, DBD 2mg/mL versus Con. ** *p* < 0.01, DBD 4mg/mL versus Con. *** *p* < 0.001, DBD 4mg/mL versus Con. ^###^ *p* < 0.001, Verapamil versus Con.

The intracellular accumulation of Rh 123 in A549 cells was measured by flow cytometry. Verapamil (50 μM) was used as a positive control. As shown in Figure 6A, DBD treatment significantly inhibited the efflux activity of P-gp at the concentration of 2 and 4 mg/mL.

### 2.8. Protein Expression of P-gp in DBD Treated A549 Cells

Western blot analysis was performed and the relative expression of P-gp in A549 cells is shown in Figure 6B, C. It was found that after 48 h of 2 mg/mL or 4 mg/mL DBD treatment, the P-gp level was significantly decreased compared with that of the Control group.

### 2.9. mRNA Expression of hENT1 and hCNT1 in DBD Treated A549 Cells

Cellular uptake of GEM is largely mediated by ENTs and CNTs. The presence of nucleoside transport activity is considered a prerequisite for cell growth inhibition and clinical efficacy of GEM. To further investigate whether the ENTs and CNTs are regulated by DBD, mRNA expression of hENT1 and hCNT1 in A549 cells was evaluated by real-time qPCR.

**Figure 7:**
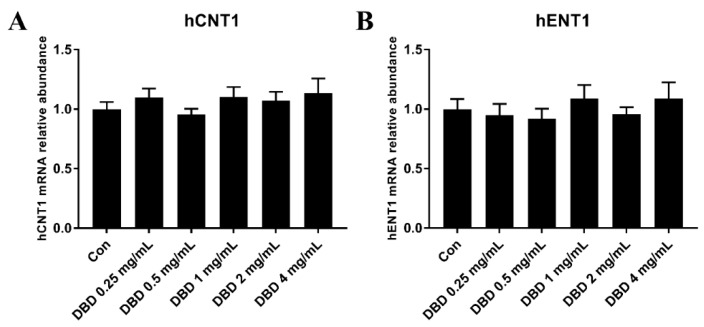
The mRNA expression of hCNT1 (**A**) and hENT1 (**B**) in DBD-treated A549 cells. Data were expressed as mean ± SD of four independent experiments.

As seen in Figure 7, after being treated with various concentrations of DBD, the level of hCNT1 and hENT1 showed no significant difference between the control group and the DBD group in A549 cells, indicating that DBD has no influence on the mRNA expression of hENT1 and hCNT1.

## 3. Discussion

According to the therapeutic guideline for NSCLC, gemcitabine-based chemotherapy is the current standard treatment for advanced NSCLC [24]. However, the chemotherapy often results in side effects and chemo-resistance [3]. In order to improve the overall survival and life quality of NSCLC patients, novel strategies are needed to enhance the chemo-sensitivity of GEM. The combination of some traditional Chinese medicines with chemotherapeutic drugs were found to have the ability to increase the efficacy and reduce the side effects of the chemotherapy [10,11,12].

In this study, a transplanted model was established by grafting LLC cells in C57BL/6J mice to test the combination effects of GEM and DBD. According to the result, co-administration of 1.8 g/kg DBD considerably augmented the anti-tumor effect of GEM in LLC tumor-bearing mice, and DBD alone presented no therapeutic effect on LLC-transplanted tumor.

To further evaluate the therapeutic effect of GEM combined with DBD, we investigated the pharmacokinetics process in vivo after the combination administration of GEM and DBD. According to the results, the C_max_, T_max_, T_1/2_, and AUC_0–t_ of dFdC and dFdU showed no significant difference between the GEM-treated group and the GEM + DBD-treated group, suggesting that DBT might not be able to regulate the activity of cytidine deaminase (CDA). Pharmacokinetic interactions of GEM and DBD were observed by a significant increase in C_max_ and AUC_0–t_ of dFdCTP, indicating that co-administration of DBD might have increased the active metabolite level of GEM in rats. The heightened dFdCTP level could be an explanation for the enhanced anti-tumor effect of GEM by DBD co-administration.

According to the literature, the pharmacokinetic interactions between traditional Chinese medicine and drugs are mainly caused by the regulation of metabolic enzymes and drug transporters [14], especially for the efflux transporter P-gp. It is known that GEM is a pro-drug and has to be phosphorylated by dCK within the tumor cells to become active [8]. Therefore, the pharmacokinetics results obtained in this study indicated that DBD could possibly increase the level of dFdCTP by regulating the activity of P-gp and dCK.

To further investigate the expression levels of P-gp and dCK in LLC tumor-bearing mice after the combination administration of GEM and DBD, our western blot and RT-PCT results confirmed the up-regulation of dCK and down-regulation of P-gp in the tumor tissue of the GEM + DBD group mice, while DBD treatment did not alter the expression of hCNTs and hENTs in A549 cells. Moreover, our rhodamine 123 accumulation result indicated the potential inhibition effect of DBD on P-gp activity.

Studies from decades ago manifested the increased sensitivity to gemcitabine in P-gp overexpression cells [25,26]. To the contrary, recent research indicated that down-regulation of P-gp expression could increase gemcitabine sensitivity [16,17,18]. Based on the fact that dCK could convert GEM into dFdCTP and P-gp could play a critical role in the acquired resistance of cancer to GEM, we believe that the co-administration of DBD alters the GEM metabolism by regulating the expression and activity of dCK and P-gp, which could be a reasonable explanation for the significantly heightened Cmax and AUC_0–t_ of dFdCTP in pharmacokinetic results.

Moreover, DBD treatment increased IL-12p70 and GM-CSF expression in mice plasma, illustrating the immune regulatory effect of DBD, while IL-12p70 and GM-CSF were recently reported to be involved in tumor immunotherapy [27,28].

Summarizing these results and perspectives, it could be deduced that the active metabolite of GEM, dFdCTP, was notably increased under the co-administration of DBD, where the up-regulated dCK and down-regulated P-gp could be convincing reasons for this. According to the reports, in vitro models have shown cross-resistance between cladribine, gemcitabine, fludarabine, and cytarabine with reduced dCK activity as the underlying determinant of resistance [29,30], which indicated the potential sensitization effect of DBD on other nucleoside analogues. On the other hand, the immunoregulation effect of DBD could be a reason for the increased anti-tumor effect of GEM + DBD mice when compared with the GEM group. The present data showed for the first time that DBD interacted with the metabolism of GEM. DBD increased dCK mRNA and protein expression in tumor-bearing mice, which may affect its enzymatic activity and its role as a potential marker of drug sensitivity in the clinical setting, indicating the potential clinical benefits of combination usage of DBD with GEM and other nucleoside analogues for the treatment of NSCLC.

## 4. Materials and Methods

### 4.1. Reagents and Materials

HPLC grade methanol and acetonitrile were purchased from Tedia Company Inc. (Fairfield, OH, USA). Analytical grade formic acid, ammonium acetate, and DMSO were obtained from Nanjing Chemical Reagent Co., Ltd. (Nanjing, China). The chemical reference substance of dFdU was purchased from Toronto Research Chemicals (Toronto, ON, Canada). dFdC hydrochloride was obtained from JARI (Lianyungang, China). dFdCMP, dFdCDP, and dFdCTP were obtained from SUNDIA (Shanghai, China). Tetrahydrouridine (THU) and dCK antibody were from Santa Cruz (sc-393099, Dallas, TX, USA). Dulbecco’s Modified Eagle Medium (DMEM) was purchased from Gibco (Grand Island, NY, USA). Fetal bovine serum (FBS) was purchased from Biological Industries (Kibbutz Beit-Haemek, Israel). P-gp antibody was purchased from Cell Signaling Technology (catalog number 13978, Danvers, MA, USA). Rhodamine 123 was purchased from Sigma-Aldrich (St. Louis, MO, USA). Verapamil hydrochloride was purchased from Aladdin (Shanghai, China). β-actin antibody and all secondary antibodies were purchased from SAB (catalog number 49294, L3012-2 and L3032-2, College Park, MD, USA).

### 4.2. Preparation and Determination of DBD

RA and RAS were procured from a local traditional Chinese medicine store in Nanjing. All our traditional Chinese medicine materials were authenticated by Dr. Yin Zhiqi at China Pharmaceutical University. Briefly, 50 g of RA and 10 g RAS all in fine powder form were weighted. RAS was put in a Soxhlet extractor, and refluxed ten times with 150 mL ethanol, the ethanol extract containing most of the essential oil of RAS was collected for later use. The RAS dreg was put together with RA, immersed in water, and decocted twice at boiling temperature for 2 h each with 480 mL of water. Then the water solutions were combined and concentrated with vacuum rotary evaporation using a 75 °C water bath to a volume of about 180 mL, then mixed with ethanol in a volume ratio of 180:540. The mixture was left to stand for 12 h, then the supernatant was collected and concentrated until all the ethanol and most of the water were evaporated. The residual was then freeze-dried to yield the solid extract about 18 g (extraction yield about 30% w/w on anhydrous basis with the water content about 2.3%). The residual was then dissolved in 60 mL distilled water and mixed with the RAS ethanol extraction, and then the ethanol was evaporated with gentle vacuum rotary evaporation using 25 °C water bath to make the DBD containing extractants of 0.3 g/mL.

The decoction product of DBD used for the study was analyzed by a validated reversed phase HPLC system (Thermo Dionex Ultimate 3000 HPLC system, Thermo Fisher Scientific, Waltham, MA, USA) using a GL Sciences InertSustain C18 column (4.6 × 250 mm, 5 μm). The following gradient system was employed: mobile phase A (5% methanol containing 0.1% formic acid) and mobile phase B (50:50 methanol: acetonitrile), 10% (*v*/*v*) B at 0 min; 80% B at 60 min; 10% B at 61 min; 10% B at 68 min. The injection volume was 20 µL. The flow rate was 1 mL/min and ultraviolet detection was performed at 320 nm with a DAD-3000 (RS) diode array UV/VIS detector (Thermo Fisher Scientific, MA, USA). A representative HPLC chromatogram of DBD is displayed in Figure S1. As shown in Table S1, the 0.3 g/mL (*w*/*v*, dry weight/water) DBD contained Z-Ligustilide (333.1 μg/mL), ferulic acid (57.8 μg/mL), ononin (75.9 μg/mL), coniferyl ferulate (2.5 μg/mL), and calycosin-7-*O*-β-d-glucoside (131.1 μg/mL).

### 4.3. Cell Culture

A549 cell line was obtained from American Type Culture Collection (ATCC). Lewis lung carcinoma (LLC) cell line was obtained from Cell Bank, Shanghai Institutes for Biological Sciences of Chinese Academy of Sciences (Shanghai, China). Cells were maintained in DMEM supplemented with 10% fetal bovine serum (FBS) and 1% penicillin/streptomycin at 37 °C with a humidified 5% CO_2_ incubator.

### 4.4. LLC Tumor Model

Male C57BL/6 mice (ethic approval number: 201904001) within 6-weeks-old were obtained from Shanghai SIPPR-Bk Lab Animal Co., Ltd. Each mouse was injected subcutaneously with LLC cells (2 × 10^6^ in 100 μL of PBS) in the right flank near the hind limb, as previously described [31]. When the subcutaneous tumors were approximately 0.3 × 0.3 cm^2^ (two perpendicular diameters) in size, mice were randomized into four groups. Mice were administrated vehicle alone (0.9% saline), GEM alone (100 mg/kg i.p., once a week), DBD alone (1.8 g/kg i.g., each day), or a combination of GEM and DBD (same as the relevant single drug group). The body weights of mice and the two perpendicular diameters (A and B) of tumors were recorded every day. The tumor volumes (V) were estimated according to the formula V = A × B^2^/2, as published previously [32]. After 12 days the mice were sacrificed.

### 4.5. Western-Blot Analysis

LLC tumor-bearing mice tumor tissue (50 mg) protein samples were obtained from a supernatant of homogenized and centrifuged tissue lysate. A549 cells were collected and protein samples were obtained from RIPA lysis buffer lysate. The samples were then separated on a 10% SDS polyacrylamide gel electrophoresis and transferred onto PVDF membranes. The membranes were blocked with 5% defatted milk in TBST for 1 h and incubated with primary antibodies of P-gp (1/1000), dCK (1/100), and β-actin (1/1000) at 4 °C overnight. The membranes were rinsed with TBST thrice and incubated with 1/2000 diluted secondary antibodies (HRP-goat anti-rabbit or HRP-goat anti-mouse) for 1 h at room temperature. The density of bands was visualized and determined by chemiluminescence. β-actin was used as an internal control for protein loading.

### 4.6. Quantitative Polymerase Chain Reaction (qPCR)

Total RNA from tumor tissue of LLC-bearing mice or A549 cells were isolated using the TRIzol extraction method. Each sample contained 0.5 µg of cDNA in 10 µL of Takara TB Green quantitative PCR (qPCR) Master Mix (Kusatsu, Japan). Glyceraldehyde-3-phosphate dehydrogenase (GAPDH) were detected and were used as endogenous controls. The PCR conditions were as follows: denatured at 95 °C for 30 s, amplified for 40 cycles with 95 °C for 15 s and 60 °C for 1 min per cycle. Melting curves were performed to investigate the specificity of the PCR reaction. Data were analyzed according to the 2^−ΔΔCt^ method, and the relative amount of each studied mRNA was normalized to the level of the target genes in the normal tissues. The primer sequences were shown in Table 3.

### 4.7. Enzyme-Linked Immunosorbent Assay (ELISA)

The serum IL-2, IL-12p70, and GM-CSF expression of LLC tumor-bearing mice were detected by mouse IL-2, IL-12p70, and GM-CSF ELISA kit (Cat No. EM002-96, EM006-96 and EM020-96, Excell, China) following the manufacturer’s instruction. Briefly, the blood was centrifuged at 3000 rpm for 5 min. Serum was separated and serum cytokine concentrations were determined in duplicate.

### 4.8. Rhodamine 123 (Rh 123) Accumulation Analysis

The intracellular accumulation of Rh 123 in A549 cells was measured by flow cytometry as previously described [33]. First, the cells were plated onto 6-well plates at a density of 10^5^/well and were then incubated with DBD or verapamil for 48 h. Then cells were exposed to Rh 123 (5 μM) at 37 °C for 1 h. After treatment, cells were trypsinized and collected, washed thrice with ice-cold PBS, and analyzed by FACS (BD Biosciences).

### 4.9. Pharmacokinetic Study in Rats

For the plasma pharmacokinetic study, 20 Sprague-Dawley (SD) rats (10 males and 10 females) were obtained from Shanghai SIPPR-Bk Lab Animal Co., Ltd. Rats were maintained under specific-pathogen-free conditions in a unidirectional airflow room at 20–24 °C and relative humidity of 30‒70% with a 12 h light/dark cycle. Rats were given filtered tap water and commercial rat chow ad libitum and allowed to acclimate to the facilities and environment for 3 days before use. Rats were randomly divided into a GEM group and a GEM combined with DBD (GEM + DBD) group. Rats in the GEM group received a tail vein bolus intravenous administration of 50 mg/kg gemcitabine. In the GEM + DMD group, DBD was co-administered at the dose of 3.6 g/kg by intra gavage administration, whereas the GEM group was treated with saline. Blood samples (about 0.2 mL) were obtained from the postorbital venous plexus before the dose (0 h) and at 2, 5, 10, 15, 30 min, 1, 4, 12, and 24 h after administration. Blood was collected in heparinized tubes spiked with tetrahydrouridine (25 μg/mL) to inhibit CDA activity. The blood samples were immediately centrifuged at 3000 rpm for 5 min to obtain plasma. All the plasma samples were stored at −80 °C until analysis.

For the PBMC pharmacokinetic study, 200 SD rats, equal number of males and females, were randomly and equally divided into 10 GEM groups and 10 GEM + DBD groups, each for one time point. The administration of GEM and DBD to each group were in the same way as the plasma pharmacokinetic study. About 4 mL blood was obtained from every rat in each group through the postorbital venous plexus before the dose (0 h) and at 5, 10, 30 min, 1, 2, 4, 8, 12, and 24 h after the administration. Peripheral blood mononuclear cells (PBMCs) were separated using Histopaque^®^-1083 density gradient (Sigma). 100 μL PBMCs were separated, and a volume of 20 µL of the cell suspension of PBMCs was removed for the analysis of protein concentrations using the BCA Protein Assay Kit (P0010, Beyotime, Shanghai, China). The amount of protein was determined for all suspensions of PBMCs and used for the calculation of dFdCTP concentration in nanogram per milligram of protein. The remaining cell suspension was processed following a previously described sample preparation method [23].

### 4.10. LC-MS/MS Based Bioanalytical Assays

Validated bioanalytical assays were used to measure dFdC and its metabolites in various biological samples of rats. The LC-MS/MS system consisted of a Thermo Dionex Ultimate 3000 HPLC system (Thermo Fisher Scientific, MA, USA) with a quaternary gradient pump, a column oven, and an autosampler, coupled to a TSQ Quantum Ultra AM triple quadrupole mass spectrometer (Thermo Fisher Scientific, MA, USA) with an electrospray ion source.

For the sample preparation, an aliquot of 50 μL plasma sample in 2 mL Eppendorf tube was spiked with 50 μL of IS solution and 50 μL of methanol, or of the corresponding standard solutions when preparing calibration and QC samples. Then 100 μL methanol was added and the samples were vortex-mixed for 3 min. The supernatant was taken into a new Eppendorf tube after centrifuging at 16,000× *g* for 10 min and evaporated to dryness under vacuum at room temperature. The residual was reconstituted with 0.2 mL of the mobile phase and centrifuged at 16,000× *g* for 5 min. The supernatant obtained was then injected for the LC-MS/MS analysis.

For dFdC and dFdU in rat Plasma, the separation was achieved on a GL Sciences Inertsil C8 column (4.6 × 150 mm, 5 μm) at 35 °C. The following gradient system was employed: mobile phase A (5% methanol) and mobile phase B (20% methanol), 0% (*v*/*v*) B at 0–1.5 min; 80% B at 2 min; 80% B at 5.5 min; 0% B at 5.7 min; 0% B at 7 min; The flow rate was 1.0 mL/min. The injection volume was 20 µL. The MS/MS conditions were optimized as follows: the spray voltage was set at 4 kV with the capillary temperature at 350 °C. Nitrogen was used as sheath (40 kPa) and auxiliary (5 kPa) gases. The mass spectrometry measurement was performed in the positive ion mode with precursor–product ion pairs for selected-reaction-monitoring of dFdC, dFdU and internal standard lamivudine at *m*/*z* 264→112, *m*/*z* 265→113 and *m*/*z* 230→112. The collision energy was 14 eV for dFdC, 13 eV for dFdU and 12 eV for lamivudine.

The LC-MS/MS method was validated on accuracy, precision, recovery, selectivity, linearity, matrix effect, and stability of dFdC and dFdU. All the calibration curves showed good linearity with correlation coefficients better than 0.9905 (Figure S2 and Table S2). As shown in Table S3, the intra-day and inter-day accuracy, precision, matrix effect and extraction recovery were validated under the limit of 15%. It showed no endogenous interference with the measurement of dFdC and dFdU in selectivity validation (Figure S4). The analytes in plasma were stable under the following conditions: 12 h at room temperature, a period of 2 weeks of storage at −80 °C, 24 h in the autosampler (4 °C) and 3 freeze-thaw cycles at −80 °C (Table S4).

For dFdCMP, dFdCDP, and dFdCTP in rat PBMC, the separation was achieved on a HyperCarb column 2.1 × 100 mm with 5 μm particles size (Thermo Fisher Scientific) using a previously described LC-MS/MS method [23]. The calibration curves showed good linearity with correlation coefficients better than 0.9985 (Figure S3). Representative MRM chromatograms were shown in Figure S5. The mass spectrometry measurement was performed in the positive ion mode with precursor–product ion pairs for selected-reaction-monitoring of dFdCMP, dFdCDP, dFdCTP and internal standard lamivudine at *m*/*z* 344→246, *m*/*z* 424→326, *m*/*z* 504→326 and *m*/*z* 230→112. The collision energy was 15 eV for dFdCMP, 12 eV for dFdCDP, 18 eV for dFdCTP, and 12 eV for lamivudine.

### 4.11. Statistical Analysis

Pharmacokinetic parameters were calculated by WinNonlin 6.2 (Pharsight, St. Louis, MO, USA). Unless otherwise noted, statistical differences/significance were determined using two-tailed Student’s *t*-test (two groups) or one-way ANOVA with post-hoc Bonferroni/Dunnett’s test (three or more groups). When the *p* value is less than 0.05, statistical differences were considered significant.

## Figures and Tables

**Table 1:** Pharmacokinetic parameters of dFdC and dFdU in rat plasma after single administration of GEM and GEM + DBD. Data are shown as mean ± SD (*n* = 10).

Parameters	dFdC		dFdU	
	GEM	GEM + DBD	GEM	GEM + DBD
C_max_ (μg/mL)	11.60 ± 2.66	13.61 ± 1.64	0.34 ± 0.07	0.31 ± 0.05
T_max_ (h)	0.32 ± 0.29	0.11 ± 0.14	4.00 ± 0	4.00 ± 0
t_1/2_ (h)	3.61 ± 0.59	3.38 ± 0.28	17.73 ± 3.85	16.89 ± 4.66
AUC_0–t_ (μg·min/mL)	2868 ± 835	2814 ± 387	300.2 ± 47.2	258.5 ± 77.9
AUC_0–∞_ (μg·min/mL)	2914 ± 866	2850 ± 397	526.7 ± 66.1	491.3 ± 50.1

**Table 2:** Pharmacokinetic parameters of dFdCTP in rat PBMC after single administration of GEM and GEM + DBD. Data are shown as mean ± SD (*n* = 10), *** *p* < 0.001 compared with the GEM group.

Parameters	dFdCTP	
	GEM	GEM + DBD
C_max_ (ng/mg protein)	2.05 ± 0.43	3.80 ± 0.79 ***
T_max_ (h)	2.40 ± 0.60	1.09 ± 0.39 ***
t_1/2_ (h)	7.57 ± 2.24	6.96 ± 2.89
AUC_0–t_ (ng·min/mg protein)	798 ± 251	1266 ± 145 ***
AUC_0–∞_ (ng·min/mg protein)	998 ± 244	1415 ± 176 ***

**Table 3:** Primer sets for quantitative RT-PCR.

Gene	Forward Primer (5′→3′)	Reverse Primer (5′→3′)	Product Length (bp)
Mdr1	GTGGGGGACAGAAACAGAGA	GAACGGTAGACAAGCGATGAG	183
dCK	GGACTCTGAAAACCAGCTTTGATT	CCAGGCTTTCGTGTTTGTCTTTA	93
GAPDH-mouse	CAAGGCTGTGGGCAAGGTCA	AGGTGGAAGAGTGGGAGTTGCTG	242
GAPDH-human	ACAACTTTGGTATCGTGGAAGG	GCCATCACGCCACAGTTTC	101
hENT1	TCTCCAACTCTCAGCCCACCAA	CCTGCGATGCTGGACTTGACCT	151
hCNT1	CATTACTGATCCGGCCCTACTT	TGGCGTAACCTCCGGTCAT	75

## Supplementary Materials

The following are available online. Figure S1: HPLC chromatogram of determination of Danggui Buxue decotion, Figure S2: Linear regression data of dFdC and dFdU in rat plasma, Figure S3: Linear regression data of dFdCTP in rat PBMC, Figure S4: Representative MRM chromatograms of dFdC and dFdU in rat plasma, Figure S5: Representative MRM chromatograms of dFdCMP, dFdCDP and dFdCTP in rat PBMC, Table S1: Concentration of active ingredients in DBD, Table S2: Linear regression data and lower limit of quantitation (LLOQ) of dFdC and dFdU in rat plasma, Table S3: Summary of precision, accuracy, recovery and matrix effect of dFdC and dFdU in rat plasma, Table S4: Stability of dFdC and dFdU in rat plasma under different conditions.
