# Supplementary material for: Danggui Buxue Decoction Sensitizes the Response of Non-Small-Cell Lung Cancer to Gemcitabine via Regulating Deoxycytidine Kinase and P-glycoprotein
Source: Molecules. 2019 May 25;24(10):2011. doi: 10.3390/molecules24102011 (PMC6572355; doi:10.3390/molecules24102011)
Supplement: Supplementary file 1 [file molecules-24-02011-s001.pdf]

Supplementary materials

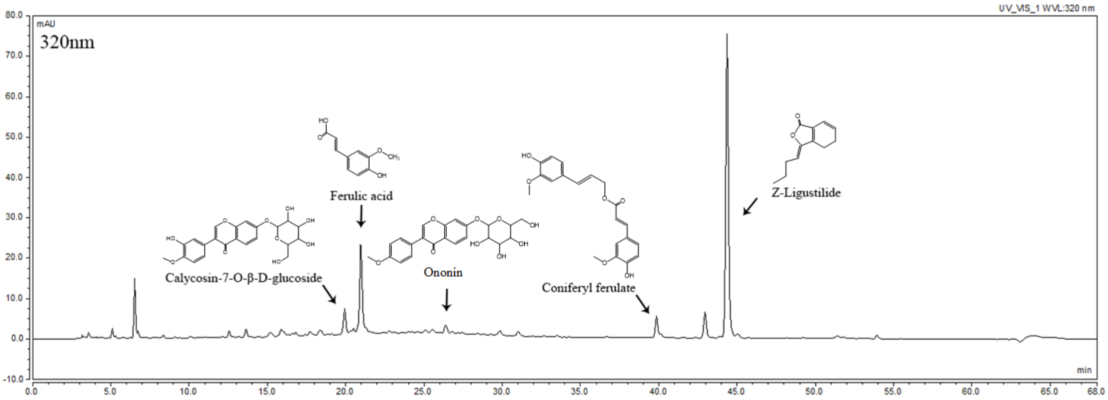

Fig. S1. HPLC chromatogram of determination of Danggui Buxue decotion. The 0.3 g/mL (w/v, dry weight/water) DBD contained Z-Ligustilide (333.1 μg/mL), ferulic acid (57.8 μg/mL), ononin (75.9 μg/mL), coniferyl ferulate (2.5 μg/mL) and calycosin-7-O-β-D-glucoside (131.1 μg/mL). The decoction product of DBD used for the study was analyzed by a validated reversed phase HPLC system using a GL Sciences InertSustain C18 column (4.6×250mm, 5μm). The following gradient system was employed: mobile phase A (5% methanol containing 0.1% formic acid) and mobile phase B (50:50 methanol: acetonitrile), 10% (v/v) B at 0 min; 80% B at 60 min; 10% B at 61 min; 10% B at 68 min. The injection volume was 20 μl. The flow rate was 1 mL/min and ultraviolet detection was performed at 320 nm with a DAD-3000(RS) diode array UV/VIS detector.

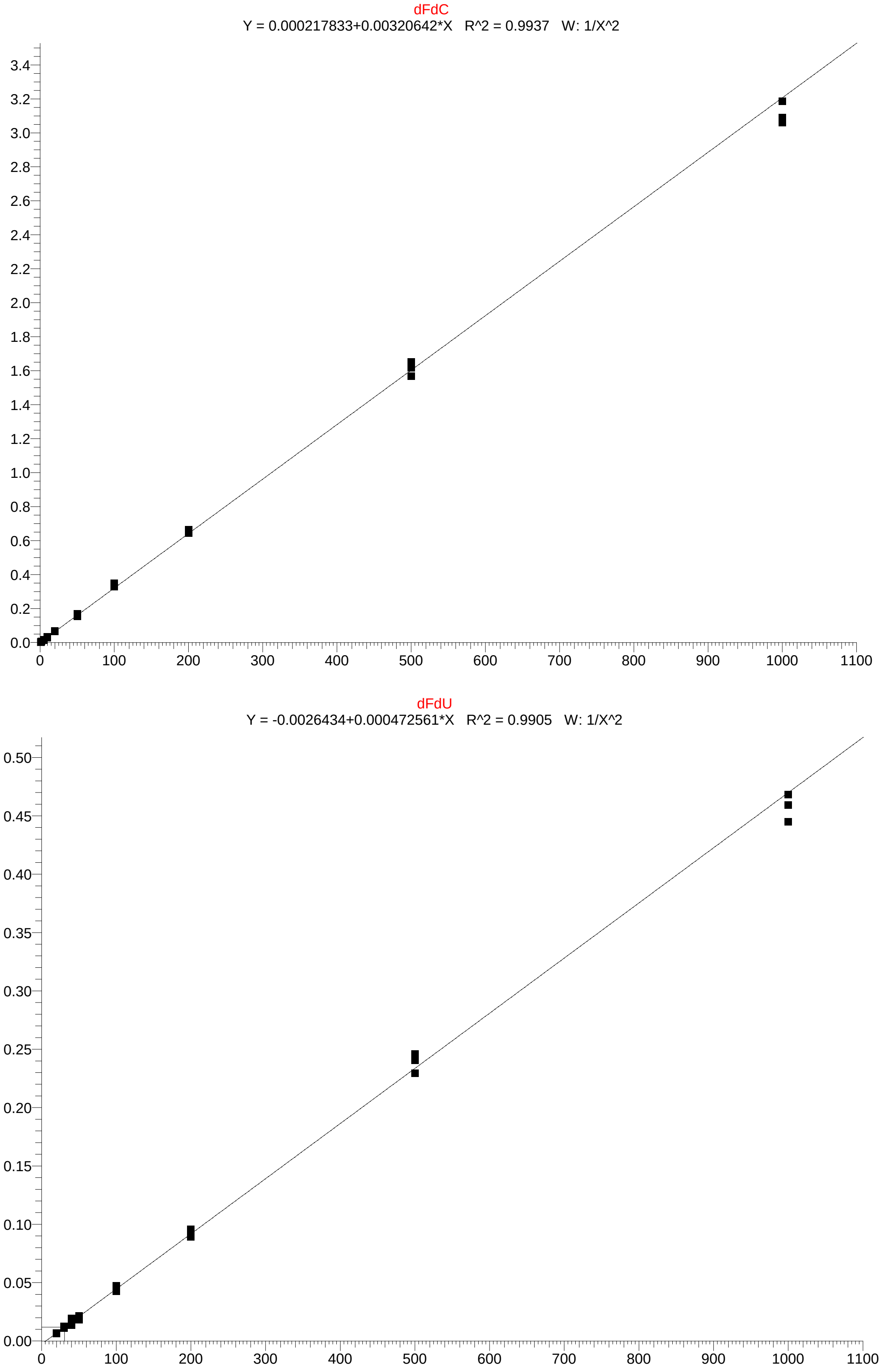

Fig. S2. Linear regression data of dFdC and dFdU in rat plasma. The linear range for dFdC was 1–1000 ng/mL, and for dFdU was 20-1000 ng/mL.

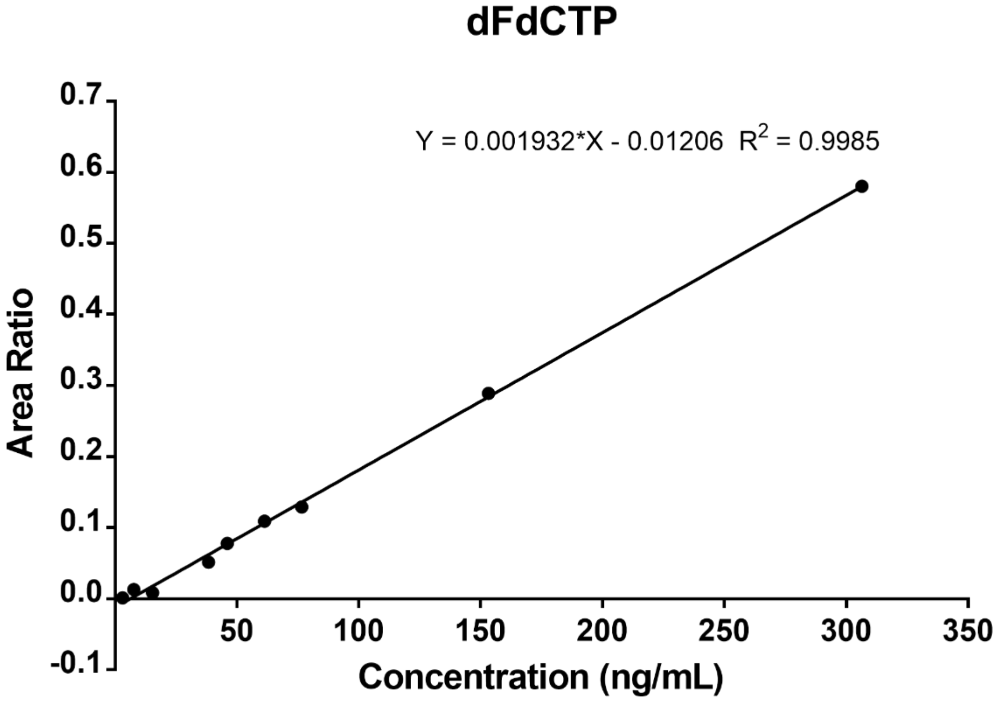

Fig. S3. Linear regression data of dFdCTP in rat PBMC. The linear range for dFdCTP was 3.06–306.45 ng/mL.

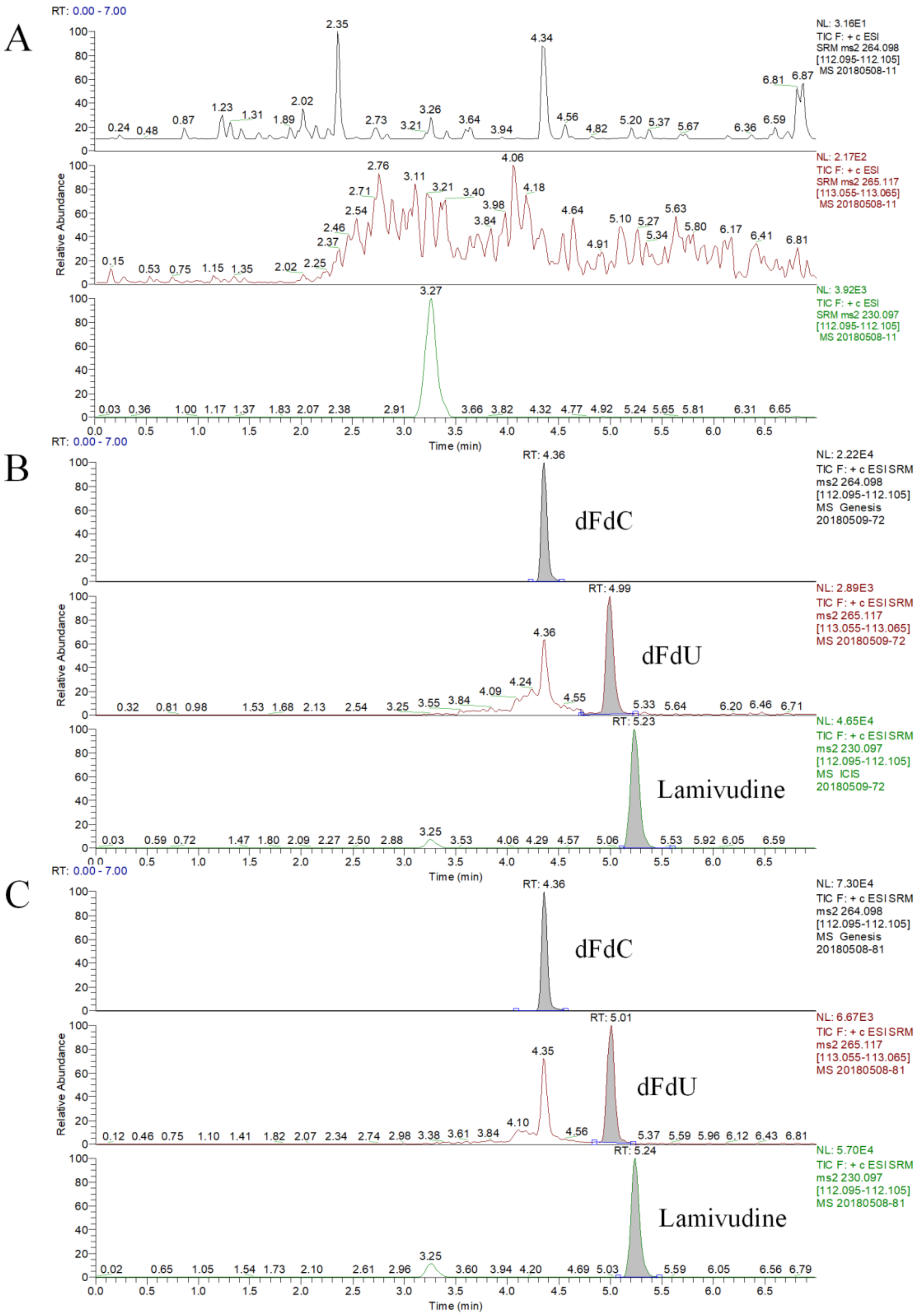

Fig. S4. Representative MRM chromatograms of dFdC and dFdU in rat plasma. (A) a blank plasma sample; (B) a blank plasma sample spiked with 50 ng/mL dFdC, 100 ng/mL dFdU and 40 ng/mL lamivudine as IS; (C) a 12 h plasma sample with 286.9 ng/mL dFdC and 254.1 ng/mL dFdU after intravenous administration of Gem in Gem treated group. MRM transitions and retention time: Lamivudine: *m/z* 230→112 at 5.23

min; dFdC: *m/z* 264→112 at 4.36 min; dFdU: *m/z* 265→113 at 5.00 min.

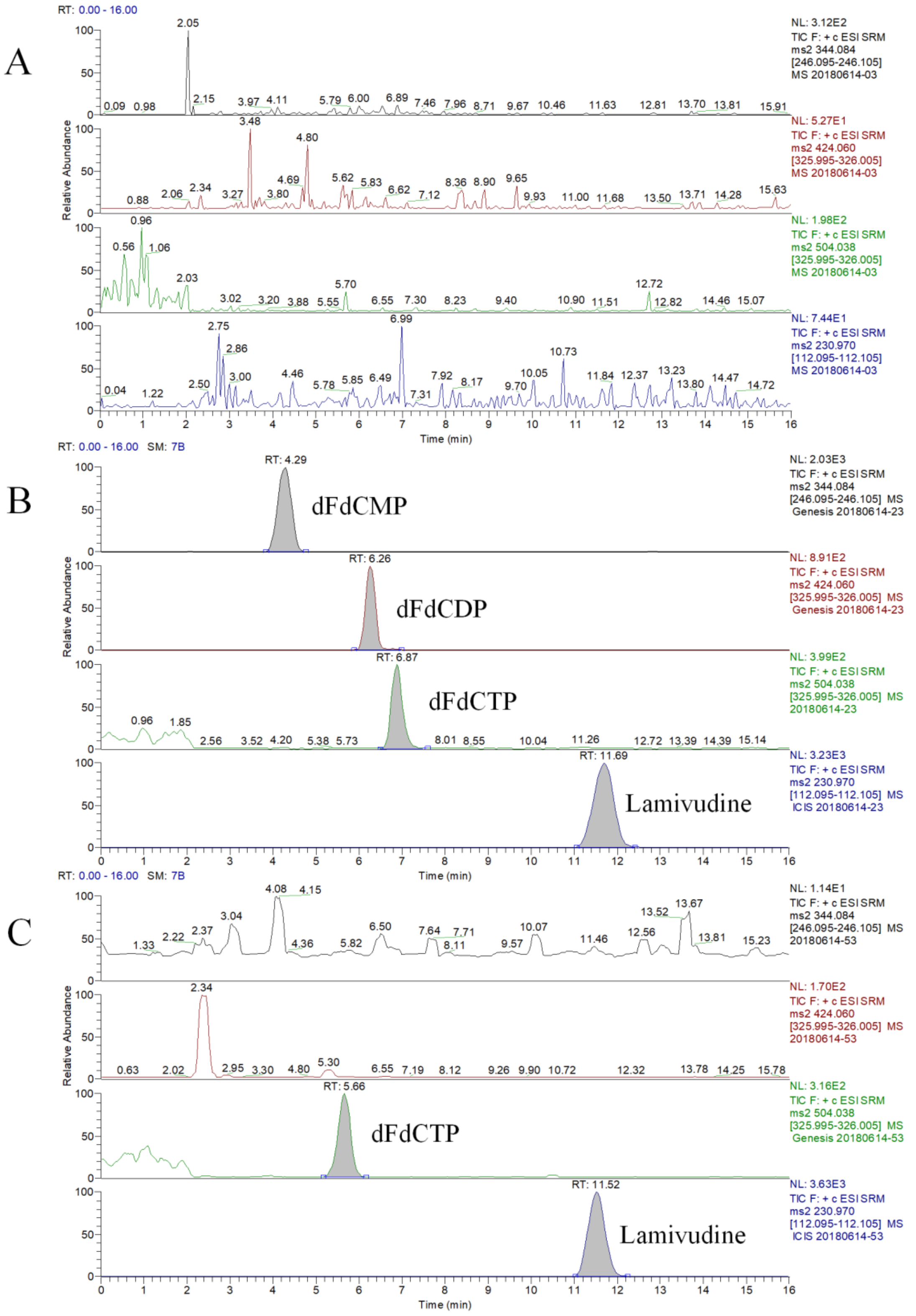

Fig. S5. Representative MRM chromatograms of dFdCMP, dFdCDP and dFdCTP in rat PBMC. (A) a blank PBMC sample; (B) a blank PBMC sample spiked with 100 ng/mL dFdCMP, 35 ng/mL dFdCDP, 60 ng/mL dFdCTP and 40 ng/mL lamivudine as IS; (C)

a 10 min PBMC sample with 62.0 ng/mL dFdCTP after intravenous administration of Gem in Gem treated group. MRM transitions and retention time: dFdCMP: *m/z* 344→246 at 4.29 min; dFdCDP: *m/z* 424→326 at 6.26 min; dFdCTP: *m/z* 504→326 at 6.84 min; Lamivudine: *m/z* 230→112 at 11.6 min

Table S1

Concentration of active ingredients in DBD.

Ingredient	Concentration (μg/mL)
Z-Ligustilide	333.1 ± 34.1
Ferulic Acid	57.8 ± 10.7
Ononin	75.9 ± 14.6
Coniferyl ferulate	2.5 ± 0.3
Calycosin-7-O-β-D-glucoside	131.1 ± 15.3

Data were shown as mean ± SD, n=3.

Table S2

Linear regression data and lower limit of quantitation (LLOQ) of dFdC and dFdU in rat plasma.

Analytes	Regression equation	Linear range (ng/ml)	R^2	LLOQ (ng/ml)
dFdC	y = 0.0032x + 0.0002178	1.0–1000	0.9937	1.0
dFdU	y = 0.0026x + 0.0004726	20–1000	0.9905	20

Table S3

Summary of precision, accuracy, recovery and matrix effect of dFdC and dFdU in rat plasma (n = 6). (intra-day: n = 6; inter-day: n = 6 series per day, 3 days)

Analytes	Concentration (ng/mL)	Precision (%, RSD)		Accuracy (%, RE)	Matrix (%)	Recovery (%)
		Intra-day	Inter-day			
dFdC	2.0	7.51	2.85	-8.17	98.63±8.81	117.31±7.13
	50	2.39	4.49	11.13	81.04±5.45	91.39±6.49
	500	2.42	4.63	2.38	86.16±6.28	85.92±4.11
dFdU	30	9.61	9.61	1.52	54.85±3.78	92.80±7.32
	100	6.78	4.75	3.50	55.35±5.39	79.55±1.68
	500	2.42	7.59	-0.26	50.77±5.30	77.32±4.54

Table S4

Stability of dFdC and dFdU in rat plasma under different conditions (*n* = 6).

Analytes	Concentration (ng/ml)	3 times Freeze thaw		12 h Room temperature		2 weeks Long-term		24 h Post-preparative	
		RE (%)	RSD (%)	RE (%)	RSD (%)	RE (%)	RSD (%)	RE (%)	RSD (%)
dFdC	2	-6.3	11.4	-10.5	13.7	-7.2	5.8	-8.6	6.5
	50	5.2	5.4	8.6	12.1	10.7	10.3	-2.2	3.4
	500	2.1	9.9	-5.9	6.5	-8.1	3.1	4.4	7.8
dFdU	30	11.5	9.5	-12.6	10.5	6.5	12.6	-10.5	6.7
	100	-8.6	12.5	8.7	5.8	-11.5	5.2	8.5	9.5
	500	7.1	6.8	9.2	8.5	8.2	6.8	7.2	2.9
